# On the Dissolving of Teeth in Solution of Sugar

**Published:** 1852-01

**Authors:** T. Linser

**Affiliations:** London Lancet.


### ARTICLE XIII.

### *On the Dissolving of Teeth in Solution of Sugar.*

Sir: The dissolution of teeth in lactic acid is known, as well as that sugar solution produces "lactic acid" by fermentation. The tooth I used for the experiment was a thoroughly sound one, without the least speck, and nineteen grains in weight. I put it in a solution of six drachms of sugar and three drachms of water, in a temperature of 32°—36°. In three weeks, the tooth lost about seven grains, with a change in the solution, and, in analyzing it, I found that only the carbonate and phosphate of lime were consumed. The part round the crown of the tooth was unchanged. The tooth lost, afterwards, when I put it in diluted hydrochloric acid, all its limy substance in the course of twenty-four hours, with a small influence of carbonic acid. Another tooth which I put in diluted hydrochloric acid lost in twenty-four hours all its limy substance, but under the influence of a great deal of carbonic acid. A tooth remained unchanged in very concentrated camphor solution.

A tooth, nine grains in weight, put in a solution of half a drachm of oxalic acid and three drachms of water, in a temperature of 32°—36°, lost, in ten days, two grains and a half of its weight. It was found with a white sediment (oxalate of lime) and weighed four grains. The enamel was found quite dissolved ; the other part of the tooth was not altered.

T. LINSER.

[*London Lancet.*

